# Supplementary material for: Chromothripsis during telomere crisis is independent of NHEJ, and consistent with a replicative origin
Source: Genome Res. 2019 May;29(5):737–49. doi: 10.1101/gr.240705.118 (PMC6499312; doi:10.1101/gr.240705.118)
Supplement: Supplemental Material [file supp_gr.240705.118_Supplemental_file_1.zip › contigs/annotated_contigs/DB37/contig.2.DB37_length_430_mean_cov_7.32558139535.docx]

**DB37_length_430_mean_cov_7.32558139535**

```
TGCCTAAGTTTTGAAGTTTGAATCTCAAATTCTTAAAATTCATTGCATCCTTTGTCCCTGACTTCATCATCTTTACTCAGCCATTTCCT
 >chrX:10502959-10503200 + E=1e-133
CAACCTTTAGTAGGACCGACACTGCCTTTGTCTATTCAAGATGATTTCGACTGAGTTGAAAGAAATGTGGAAATCATGGAAGGAAAGAA

GATGAGGAGTGTCTACTAGGGGAGTAGTTCCCATTCTGGCCTACACATGGCAATCACCTGGA|T|TTGGTATCTAGTTAGTCTAGCATT
                                                               >chrX:10554631-10554821
CATTAGCTAATGATGTCCTTTGGTATTTATTAAAGTCACCACAGCATGGGGGGACTTTATGTTTAGGTTTTGCCTAAGAGTTAGCTTAT
+ E=7e-103
CTGCTTCTTGTGCTAACAGGGCTGTTGCTGCTAGGGCCCTTAGACATGCAGGCCAGCCTTTGGAAACCCCGTCTAG
```
